# Analysis of the conserved protective epitopes of hemagglutinin on influenza A viruses

**DOI:** 10.3389/fimmu.2023.1086297

**Published:** 2023-02-17

**Authors:** Chenchen Jiao, Bo Wang, Pucheng Chen, Yongping Jiang, Jinxiong Liu

**Affiliations:** State Key Laboratory of Veterinary Biotechnology, National Poultry Laboratory Animal Resource Center, Harbin Veterinary Research Institute, Chinese Academy of Agricultural Sciences, Harbin, China

**Keywords:** influenza A viruses, hemagglutinin, broadly neutralizing antibodies, conserved protective epitopes, universal influenza vaccines, therapeutic agents

## Abstract

The conserved protective epitopes of hemagglutinin (HA) are essential to the design of a universal influenza vaccine and new targeted therapeutic agents. Over the last 15 years, numerous broadly neutralizing antibodies (bnAbs) targeting the HA of influenza A viruses have been isolated from B lymphocytes of human donors and mouse models, and their binding epitopes identified. This work has brought new perspectives for identifying conserved protective epitopes of HA. In this review, we succinctly analyzed and summarized the antigenic epitopes and functions of more than 70 kinds of bnAb. The highly conserved protective epitopes are concentrated on five regions of HA: the hydrophobic groove, the receptor-binding site, the occluded epitope region of the HA monomers interface, the fusion peptide region, and the vestigial esterase subdomain. Our analysis clarifies the distribution of the conserved protective epitope regions on HA and provides distinct targets for the design of novel vaccines and therapeutics to combat influenza A virus infection.

## Introduction

Influenza A viruses are negative-sense RNA viruses, belongs to the family *Orthomyxoviridae*. Their genome consists of eight single-stranded negative-sense RNA fragments, that encode 10 essential proteins. Currently, 18 different hemagglutinin (HA) (H1–H18) and 11 different neuraminidase (NA) (N1–N11) subtypes have been identified or detected (1). Influenza A viruses evolve rapidly and can cause pandemics and epidemics of acute respiratory disease in domestic poultry, lower mammals, and humans, continuously challenging the poultry industry and human health (2). According to the World Health Organization (WHO), annual influenza epidemics result in an estimated about 3 to 5 million cases of severe illness and 290,000 to 650,000 respiratory deaths worldwide (3). Influenza A viruses have also caused pandemics, including the 1918 H1N1, 1957 H2N2, 1968 H3N2 and 2009 H1N1 pandemics, which caused millions of human deaths (4–7). Occasionally, zoonotic influenza A subtypes, such as H5Nx and H7N9, also infect humans through cross-species transmission, with a mortality rate of up to 52% (8).

Vaccination remains the best strategy for preventing influenza infections. Currently, vaccines are available against seasonal influenza viruses, the vaccines contain either three (trivalent) or four (tetravalent) influenza virus components, and are formulated every year based on worldwide influenza surveillance (9). However, the effectiveness of seasonal influenza vaccines is often quite low, only 10%–60% for the influenza seasons from 2004 to 2020 (10). Two types of influenza antiviral drugs that target the viral membrane protein (M2) ion channel and inhibitors of NA also have been approved for prophylaxis and therapy. However, the use of these antivirals is still limited (11). Therefore, a universal influenza vaccine that can elicit more broadly cross-reactive and long-term protection, and novel therapeutic agents would be highly desirable. Since HA is the most important and abundant surface glycoprotein of influenza viruses and the target of almost all neutralizing antibodies (12), the HA protein is a major target for the development of universal influenza vaccine and therapeutic agents.

Over the last 15 years, numerous broadly neutralizing antibodies (bnAbs) that cross-react and neutralize a wide range of subtype HAs of influenza viruses have been isolated from B lymphocytes of human donors and mouse models, and the epitopes recognized by these antibodies have mapped through the use of escape mutants and Cryo-electron microscopy. These works have figured out the conserved protective epitope region of HA, and provide hope for development of universal influenza vaccines and new targeted therapeutic agents. Multiple efforts have therefore been made to develop broad-spectrum, universal vaccines, such as sequential vaccination with chimeric HA (13, 14), and HA stem-based immunogens (15–17). At the same time, several bnAbs themselves have been used as passive immunotherapy (18). In addition, guided by structural knowledge of the interactions and mechanism of bnAb, series of therapeutic agents such as small proteins, peptides and molecules have been designed to mimic the function of bnAb (19–23). Here, we analyzed and summarized the antigenic epitopes and functions of more than 70 kinds of bnAbs reported since 1980s. Our analysis clarifies the distribution of the conserved protective epitope regions on HA and provides new insights for the design of novel vaccines and therapeutics against influenza A virus infections.

## Overview of the HA protein

**Figure 1:**
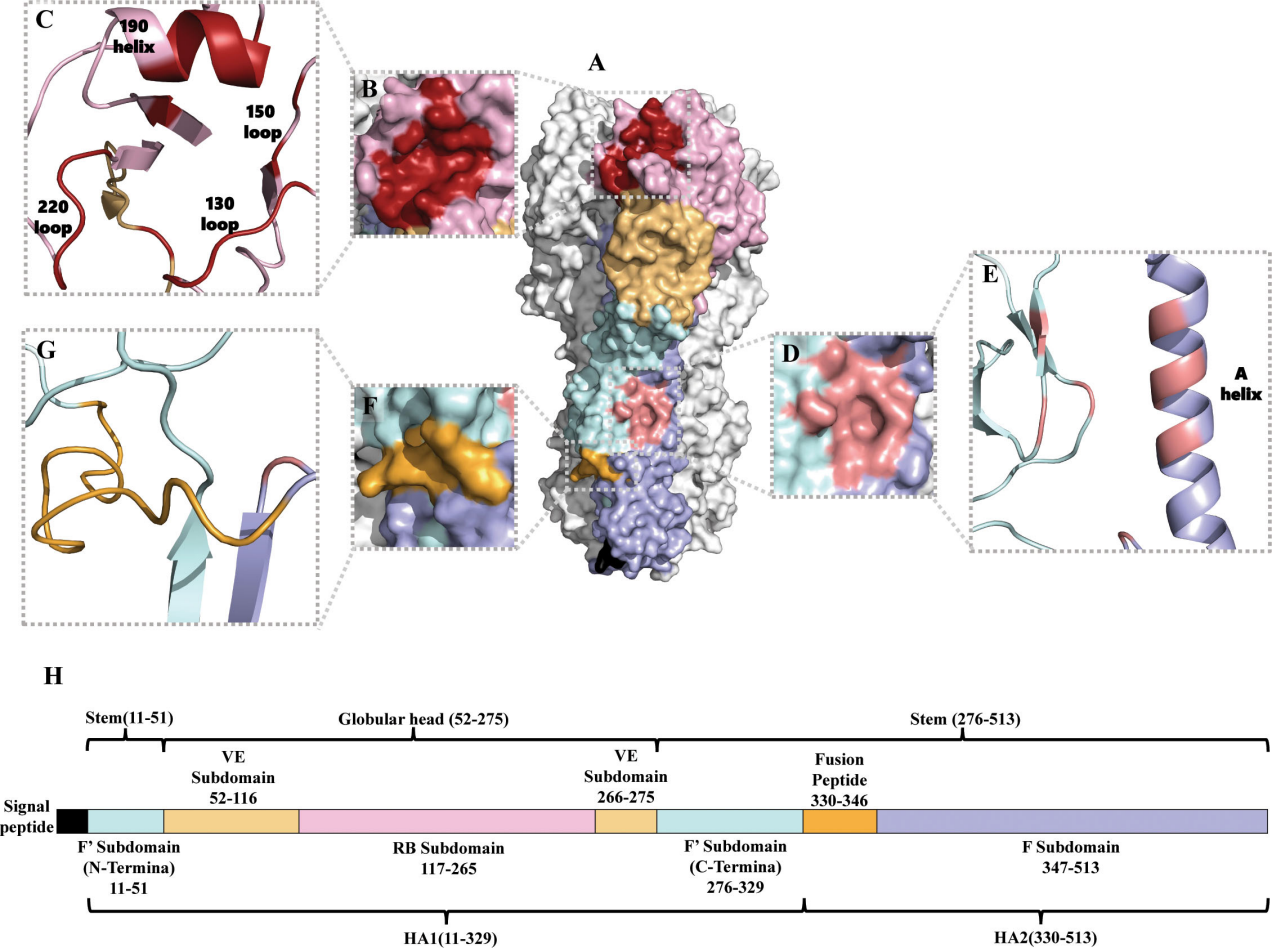
Structure of the HA of influenza A virus. **(A)** Protein molecular model of H3 HA (A/Aichi/2/68). The RB subdomain is shown in pink, the RBS in dark red, the VE subdomain in yellow, the F’ subdomain in bright blue, the F subdomain in light purple, the fusion peptide in orange-yellow, and the hydrophobic groove region in orange-red (29). **(B, C)** are the RBS surface and cartoon representation, respectively (30). **(D, E)** are the hydrophobic groove region surface and cartoon representation, respectively (31). **(F, G)** are the fusion peptide surface and cartoon representation, respectively (29). **(H)** Amino acid sequence of HA (H3 numbering) (29).

The structure of HA was identified in 1981 (24). Although the amino acid sequence homology of different subtype HAs can be as low as about 40%, HA always adopts the same protein folding and its architecture is highly conserved (25). However, the surface properties and glycosylation patterns of HA vary extensively between influenza subtypes. Influenza A viruses are divided into two phylogenic groups based on their HA, group 1 (H1, H2, H5, H6, H8, H9, H11, H12, H13, H16, H17 and H18 subtypes) and group 2 (H3, H4, H7, H10, H14, and H15 subtypes) (26, 27). Group 1 HAs has similar stem structures, whereas group 2 HAs display intra-group similarity in the stem (28). Mature HA is a trimer composed of three identical monomeric subunits (24) (**Figure 1A**). Every monomeric HA is synthesized as an immature single polypeptide chain (HA0) in the endoplasmic reticulum, and is cleaved at its cleavage site by host cell proteases to yield two subunits, HA1 and HA2, which are linked *via* a single disulfide bond (32). Each HA monomer subunit is divided into a head domain and a stem domain. The membrane-distal globular head domain composed of HA1, and contains the receptor-binding (RB) subdomain and the vestigial esterase (VE) subdomain (**Figures 1A, B, C, H**). The membrane-proximal stem domain is primarily composed of HA2 with some HA1 residues, and contains the F’ subdomains, the F subdomain, and the fusion peptide subdomain (33) (**Figures 1A, D–H**). The head domain mediates attachment of the virus to host cell surface receptors, and the stem domain mediates liberation of the viral genome into the cytoplasm through membrane fusion.

The receptor-binding site (RBS) is a shallow hydrophobic pocket at the tip of the RB subdomain, and comprises four secondary structural elements: the 130-loop, the 150-loop, the 190-helix, and the 220-loop (**Figures 1A–C**). Several key residues in the base of the pocket that interact with sialic acid (SA) are conserved, including W153, H183, L194, and Y195 (34). HA binds to the host cell *via* RBS recognizing receptors–glycolipids or glycoproteins containing terminal SA moieties with van der Waals interactions and initiates membrane fusion (35–37). Most regions of the RB subdomain besides the RBS are highly variable and prone to antigenic drift. The VE subdomain is located at the junction between the RB subdomain and the stem domain, but their functions are not well-known. The sequence of the VE subdomain is highly conserved within subtypes, and variable between different subtypes (29, 38).

The stem domain anchors HA in the viral envelope. After HA binding to the host cell receptor, endocytosis transports the influenza viral particle to the endosome, where the low pH triggers conformational changes in HA2 that mediate fusion of the viral and endosomal membranes and release of the viral genome into the cytoplasm, establishing the onset of the replication cycle (39). During this process, the HA structure is dynamic and undergoes spontaneous and reversible transitions between multiple conformations (40). The stem domain of HA is also functionally conserved, with much less sequence variation across strains and subtypes.

### Conserved protective epitopes on the head domain of HA

Despite the higher variation in the HA head domain, a series of bnAbs capable of binding and neutralizing multiple subtypes or subtype-specific influenza A virus were isolated. Their protective epitopes on the HA head domain are mainly concentrated in three regions: the RBS pocket and its surrounding area, the VE subdomain, and the occluded epitope region, which is hidden in the HA monomer interface of HA1. Anti-head bnAbs generally inhibit virus attachment to the host cell receptor, thereby blocking viral entry (41–50). However, some bnAbs targeting the VE subdomain and the occluded epitope can inhibit membrane fusion (51–53) or mediate Fc-Fcγ receptor (Fc-FcγR) interactions, antibody dependent cell-mediated cytotoxicity (ADCC) or complement dependent cytotoxicity (CDC) (54–58).

#### Epitopes of the RBS pocket and surrounding area

Residues at the rim of the RBS pocket are highly conserved, and the reactive breadth of bnAbs targeting the RBS is limited by the region of epitopes beyond the RBS pocket. Most of these antibodies show hemagglutination-inhibition (HAI) activity (41, 42, 50, 59–67), and inhibit viral entry by preventing HA binding to host receptors (41–50). They may also prevent HA conformational changes during membrane fusion by cross-linking neighboring subunits of the HA trimer, as has been reported for bnAbs HC63 (63, 68). To date, there have been no reports of anti-RBS bnAbs that can mediate Fc-FcγR responses.

**Figure 2:**
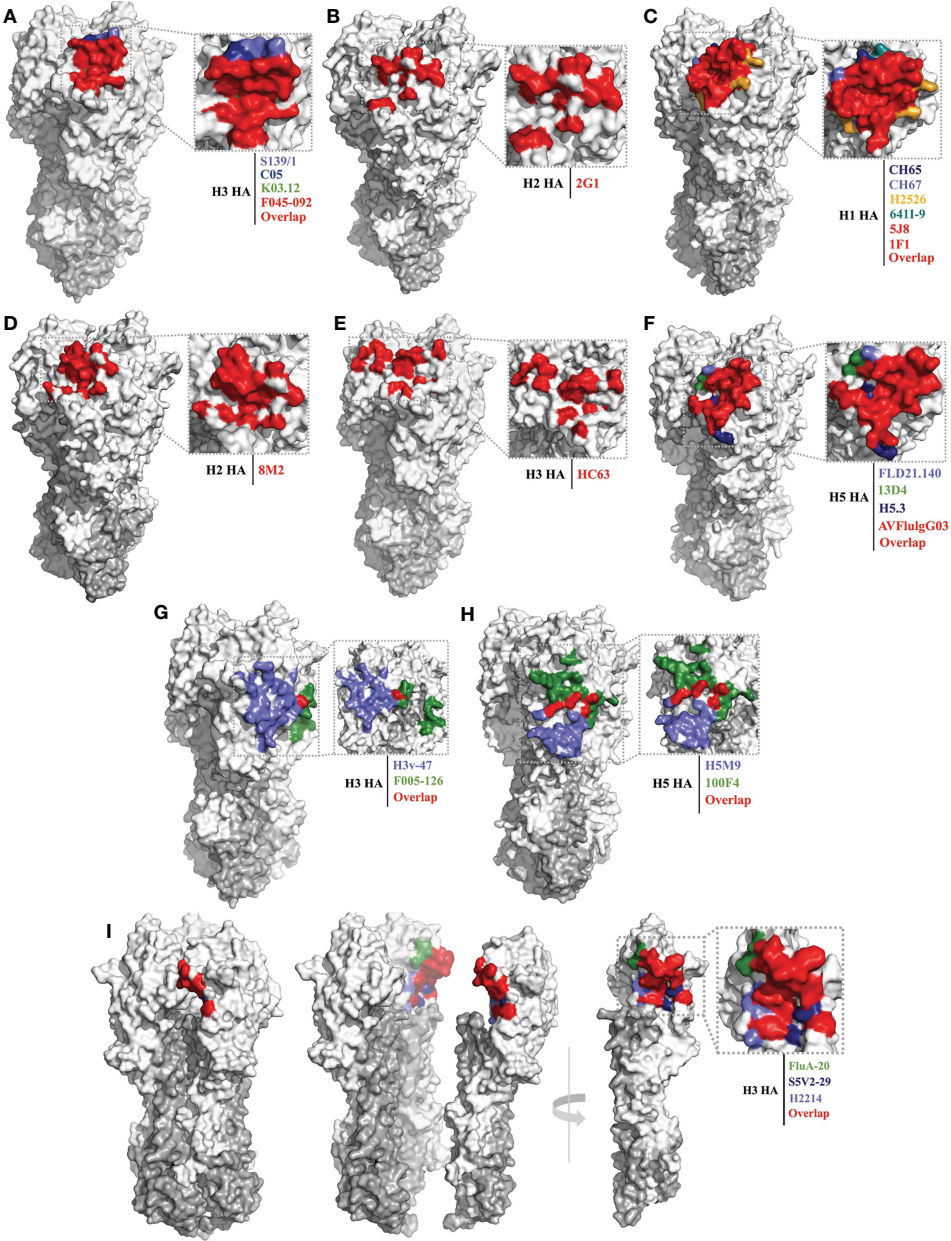
Conserved epitopes of the HA head domain. HA1 is shown in white, HA2 in gray. **(A, B)** are epitope footprints of cross-subtype bnAbs binding to the RBS. **(A)** H3 HA (PDB ID: 4FNK) as a model, the epitope of F045-092 (PDB ID: 4O58) is all overlapped shown in red, the non-overlapping residues are shown in light purple (S139/1, PDB ID:4GMS), dark blue (C05, PDB ID:4FQR), and green (K03.12, PDB ID: 5W08), respectively. **(B)** H2 HA (PDB ID: 4HLZ) as a model, the epitope of 2G1 (PDB ID: 4HG4). **(C–F)** are epitope footprints of subtype specific bnAbs binding to the RBS. **(C)** H1 HA (PDB ID: 4M4Y) as a model, the epitopes of 5J8 (PDB ID:4M5Z) and 1F1 (PDB ID: 4GXU) are overlapped in red. The non-overlapping residues are shown in dark blue (CH65, PDB ID: 5UGY), light purple (CH67, PDB ID: 4HKX), yellow (H2526, PDB ID: 4YJZ), blue-green (641I-9, PDB ID: 4YK4), respectively. **(D)** H2 HA (PDB ID: 4HLZ) as a model, the epitope of 8M2 (PDB ID: 4HFU). **(E)** H3 HA (PDB ID: 4FNK) as a model, the epitope of HC63 (PDB ID: 1KEN). **(F)** H5 HA (PDB ID: 4MHH) as a model, the epitope of AVFlulgG03 (PDB ID: 5DUP) is overlapped in red. The non-overlapping residues are shown in light purple (FLD21.140, PDB ID: 6A67), green (13D4, PDB ID: 6A0Z), and dark blue (H5.3, PDB ID: 4XNM), respectively. **(G, H)** are epitope footprints of bnAbs binding to the VE subdomain. **(G)** H3 HA (PDB ID: 4FNK) as a model, non-overlapping residues are shown in light purple (H3v-47, PDB ID: 5W42) and green (F005-126, PDB ID: 3WHE). **(H)** H5 HA (PDB ID: 4MHH) as a model, non-overlapping residues are shown in light purple (H5M9, PDB ID: 4MHH) and green (100F4, PDB ID:5DUR). **(I)** Epitope footprint of bnAbs binding to the occluded epitope region of the HA monomers interface. H3 HA (PDB ID: 2VIU) as a model, non-overlapping residues are shown in green (FluA20, PDB ID: 6OCB), dark blue (S5V2-29, PDB ID:6E4X), and light purple (H2214, PDB ID: 6E56), respectively.

Of the bnAbs that bind to the RBS pocket, the following seven possess cross-group or cross-subtype binding or neutralizing activities: S139/1 (41), C05 (42), F045-092 (60), K03.12 (69), 2G1 (59), FE17 (70) and 12H5 (71) (**Table 1**). S139/1, C05, and F045-092 can neutralize both group 1 and group 2 viruses *in vitro*. S139/1 provides heterosubtypic protection against H1N1 and H3N2 influenza virus passive immunization of mice (41, 72). C05 completely protects mice from a lethal challenge with H1N1 or H3N2 virus (42). F045-092 possesses broadly binding activity against H3 viruses that across five decades (1963–2011), also neutralized H1N1, H2N2, and H5N1 viruses (43, 60). The antibodies K03.12 and 2G1 also show broad binding activity against both group 1 and group 2 viruses (69). 2G1 was isolated from 1957 H2N2 pandemic healthy donors, inhibits the 2006 swine H2N3 influenza virus, and protects mice from a lethal challenge with H2N2 viruses (59, 73). The epitope footprints of these bnAbs are mainly concentrated within the RBS pocket, having little to no contact with the surrounding variable positions (**Figures 2A, B**). These bnAbs commonly insert a single heavy-chain complementarity-determining region (CDR) loop with hydrophobic residues into the RBS pocket.

**Table 1:** Characteristics of broadly neutralizing antibody binding to the HA head domain.

Location	Antibody name	Binding breadth *in vitro*	Neutralizing breadth *in vitro*	Protection breadth *in vivo*
RBS	S139/1^M^ (41, 72)	H1, H2, H5, H6, H9, H13, H16/H3	H1, H2, H13, H16/H3	H1/H3
	C05^H^ (42)	H1, H2, H9, H12/H3	H1, H2, H9/H3	H1/H3
	F045-092^H^ (43, 60)	H1, H2, H5, H13/H3	H1, H2, H5/H3	–
	K03.12^H^ (69)	H1/H3	–	–
	2G1^H^ (59, 73)	H2/H3	H2	H2
	FE17^H^ (70)	H1, H5	H1, H5	H1, H5
	12H5^M^ (71)	H1, H5	H1, H5	H1, H5
	1F1^H^ (47, 62)	H1	H1	H1
	5J8^H^ (44, 61)	H1	H1	H1
	CH65^H^ (45, 74)	H1	H1	–
	CH67^H^ (45)	H1	H1	–
	H2526^H^ (46)	H1	NO	–
	641I-9^H^ (46)	H1	H1	–
	3D11^M^ (75)	H1	H1	H1
	8M2^H^ (59, 73)	H2	H2	H2
	8F8^H^ (59, 73)	H2	H2	H2
	HC63^M^ (47, 63)	H3	–	–
	A2.91.3^M^ (48, 64)	H3	H3	–
	AVFlulgG03^H^ (65, 76, 77)	H5	H5	H5
	FLD21.140^H^ (77, 78)	H5	H5	H5
	13D4^M^ (49, 66)	H5	H5	H5
	HAb21^M^ (50)	H5	H5	–
	H5.3^H^ (79, 80)	H5	H5	–
	CR8033^H^ (81)	B	B	B
VE subdomain	PR8-23^M^ (82)	H1	H1	–
	H3v-47^H^ (57)	H3	H3	H3
	F005-126^H^ (51)	H3	H3	–
	A2.4.1^M^ (48, 64)	H3	H3	–
	H5M9^M^ (83, 84)	H5	H5	H5
	9F4^M^ (52, 85, 86)	H5	H5	H5
	HA-7^M^ (53)	H5	H5	H5
	100F4^H^ (76, 87, 88)	H5	H5	H5
	4F5^H^ (89)	H5	H5	H5*
	1H5^M^ (58)	H7	NO	H7
	1H10^M^ (58)	H7	NO	H7
	CR8071^H^ (81)	B	B	B
HA monomers interface	FluA-20^H^ (54)	H1, H2, H5, H6, H8, H9, H11, H12/H3, H4, H7, H10, H14, H15	NO	H1, H5/H3, H7
	S5V2-29^H^ (55)	H1, H2, H9/H3, H4, H7, H14	NO	H1/H3
	H2214^H^ (55)	H1, H2/H3, H4, H14	NO	H1/H3
	8H10^M^ (56)	H3, H4	–	H3
	FL-1066^M^ (56)	H3, H4	–	–
	H7-200 ^H (^ 90)	H7, H15	NO	H7
	H7.5^H^ (91, 92)	H7	H7	–

^H^ human antibody; ^M^ murine antibody; B, influenza B viruses; NO, no activity; -, no information; RBS, receptor-binding site; VE subdomain, vestigial esterase subdomain.

Binding breadth in vitro, cross-react with expressed different HA proteins or viruses *in vitro*.

Neutralizing breadth in vitro, effectively neutralize and cross-neutralize different influenza viruses in cells.

Protection breadth in vivo, effectively preventing and/or therapeutic efficacy against influenza virus infection in mouse animal models, with an exception “*” chicken embryo used as model.

Compared with the above-mentioned bnAbs, more bnAbs with subtype-specific reactivity have been reported and identified, including: the H1 subtype-specific antibodies 1F1 (62), 5J8 (61), CH65 (74), CH67 (45), H2526 (46), 641 I-9 (46) and 3D11 (75); the H2 subtype-specific antibodies 8M2 and 8F8 (59); the H3 subtype-specific antibodies HC63 (63) and A2.91.3 (64); and the H5 subtype-specific antibodies AVFlulgG03 (65), FLD21.140 (78), 13D4 (66), HAb21 (50), and H5.3 (79) (**Table 1**). The epitope footprints recognized by these bnAbs are also concentrated in the RBS pocket, but have more contact with the surrounding variable residues beyond the outer edges of the RBS pocket compared with the cross-subtype bnAbs (**Figures 2C–F**). This is because the surface area of the antigenic epitopes recognized by these antibodies (1200–1500 Å) is generally larger than that of the RBS pocket (800 Å) (93). Most of these subtype-specific bnAbs insert a single CDR loop into the RBS pocket, with more CDRs contacting the variable residues outside the RBS pocket; hence, these bnAbs have limited reactivity breadth (42).

The 1F1 antibody was isolated from a 1918 influenza pandemic survivor, aged 91–101 years (2–12 years in 1918), inhibits and neutralizes human H1 viruses (1918, 1930, 1943 and 1977 isolate strains), and protects mice from lethal challenge with 1918 H1 virus (47, 62). Antibody 5J8 has broadly neutralizing activity against 20th century H1N1 viruses and the 2009 pandemic H1N1 (44, 61). Antibodies CH65 and CH67 have naturalized seasonal H1 strains across three decades (1977–2007/2009) (45, 74). The H1 subtype-specific antibodies 1F1, 5J8, CH65, CH67, H2526, and 641I-9 approach the HA head from different directions and have somewhat different peripheral contact footprints outside the RBS pocket (**Figure 2C**).

Like 2G1, antibodies 8M2 and 8F8 were isolated from 1957 H2N2 pandemic healthy donors; however, 8M2 and 8F8 only react with human H2N2 viruses and a swine H2N3 strain (59, 73) (**Figure 2D**). Antibody HC63 was the earliest reported bnAb targeting the HA head in 1987; it reacts with most of the H3N2 viruses isolated between 1968 and 1982 (63). The epitope of HC63 is similar to that of 1F1 (47), but HC63 simultaneously binds two HA monomers (**Figure 2E**), effectively cross-linking them and blocking the pH-induced conformational changes in HA that drive membrane fusion (68).

Antibodies AVFluIgG03, FLD21.140, 13D4, HAb21, and H5.3 have widely cross-neutralizing activity with different clade of H5N1 viruses, and 13D4 protects mice against lethal challenge with H5N1 viruses of clades 1, 2.1, 2.2, and 2.3, even at the stage of infection when H5N1 virus has disseminated beyond the pulmonary system (66) (**Table 1** and **Figure 2F**). Antibody CR8033, which was isolated from volunteers vaccinated with the seasonal influenza vaccine, protects mice against lethal challenge with either of the Yamagata and Victoria lineages of influenza B viruses; its epitope also targets the RBS pocket (81).

Till now, a series of viral attachment inhibitors have been developed targeting RBS, such as PAA-YDS (94, 95), 6SL-PAMAM (96), S2(1–5) (97), A22 (98), D-26 (99, 100). Some of these inhibitors have been shown to protect mice from influenza virus infection, such as PAA-YDS and A22 (94, 95, 98).

#### Epitopes of the VE subdomain

BnAbs binding the VE subdomain only possess subtype-specific neutralizing activity. They include: the H1 subtype-specific antibody PR8-23 (82); the H3 subtype-specific antibodies H3v-47 (57), F005-126 (51), and A2.4.1 (64); the H5 subtype-specific antibodies H5M9 (83), 9F4 (52), HA-7 (53), 100F4 (101), and 4F5 (89); and the H7 subtype-specific antibodies 1H5 and 1H10 (58) (**Table 1**). Their epitope footprints are mainly located on the VE subdomain, but some extend into the RB subdomain (**Figures 2G, H**). These bnAbs can play a role in inhibiting virus binding to the host receptor (89) or membrane fusion (51–53), and may possess dual functions, as is the case with antibody H5M9 (83, 84). Some of them can block viral egress from infected cells (57), and mediate Fc-FcγR responses (ADCC) (57, 58).

Antibody H3v-47 exhibits potent neutralizing activity against multiple human and swine H3N2 viruses that circulated from 1989 to 2014. The H3v-47 epitope spans the VE and RB subdomains (57). Antibody F005-126 can broadly neutralize H3N2 viruses, binds to the VE subdomain (site R) and RB subdomain (site L), spans a cleft formed by two HA monomers in the HA trimer, and cross-links them (51) (**Figure 2G**).

Antibody H5M9 can neutralize different clades of H5N1 viruses (Clades 0, 1, 2.3.4, and 7), and protects mice from lethal H5N1 viral challenge both prophylactically and therapeutically *in vivo* (83, 84) (**Figure 2H**). Antibody 9F4 can neutralize H5N1 viruses of clade 1, 2.1, 2.2, 2.2.2, 2.3.2.1a, and 2.3.4 *in vitro*, and neutralizes H5N1 viruses of clade 2.2.2 *in vivo*. Antibodies HA-7 and 100F4 can potently inhibit or neutralize multiple clades of H5N1 viruses, and completely protect mice from lethal challenges of H5N1 (53). Antibody 100F4 also protects mice from lethal challenges of H5N6 and H5N8 viruses (87, 88). HA-7 specifically targets the VE subdomain and does not inhibit virus binding to the host cell receptor but does affect membrane fusion (53).

Antibodies 1H5 and 1H10 can bind to a wide range of H7 strains, but lack HAI and neutralizing activity *in vitro*. Both antibodies can engage Fc-FcγR responses, and provide protection *in vivo* upon passive transfer in the mouse model (58). Antibody CR8071 also target the VE subdomain of influenza B viruses, can protect mice against lethal challenge from the Yamagata and Victoria lineages, but is less potent *in vivo* than CR8033, and lacks HAI activity (81).

#### Epitopes of the occluded epitope region of the HA monomer interface

During the adsorption and endocytosis of influenza virus, HA can undergo spontaneous and reversible transitions between multiple conformations. Acidification and receptor binding can shift the dynamic equilibrium of HA conformation (40). The interface, occluded at the contact surface between the HA monomers of the head domain, can then be exposed to antibodies. Antibodies bind to these temporarily exposed epitopes, disrupt the HA trimeric structure and affect influenza virus replication (54). Early to 1993, Yewdell and colleagues demonstrated that the epitope of murine antibody Y8-10C2 is located at the interface of adjacent subunits of the HA head of H1 (102), but the reactive range was not identified. More recently, several bnAbs targeting this region were identified, including antibodies FluA-20 (54), S5V2-29 (55), H2214 (55), 8H10 (56), FL-1066 (56), H7-200 (90) and H7.5 (91) (**Table 1**). Most of these antibodies possess cross-group HA binding activity, and generally lack neutralizing activity *in vitro*, but confer robust protection *in vivo* against multi-subtype lethal virus challenge by mediating Fc-FcγR responses (ADCC or CDC) (54–56). The epitope footprints recognized by FluA-20, S5V2-29, H2214, 8H10, and FL-1066 are located in a similar region in the contact surface between the HA monomers of the head domain (**Figure 2I**).

Antibody FluA-20 shows extraordinary reactive breadth and affinity for recombinant HA trimers from subtypes H1 through H15, except for H13, and protects mice from lethal challenge with group 1 and group 2 viruses when as prophylaxis or therapy. FluA-20 rapidly disrupts HA trimers, inhibits the cell-to-cell spread of virus, and mediates ADCC activity *in vivo* (54). Antibodies S5V2-29 and H2214 can also bind multiple HAs of group 1 and group 2 viruses, and provide protection against lethal challenges with H1N1 and H3N2 viruses in mice (55). Antibodies 8H10 and FL-1066 possess broad reactivity with historical H3 HAs across over 30 years, and can bind a representative H4 (56). The epitope of antibody H7.5 also includes residues in the inter-HA head contact region, which allows H7.5 to simultaneously bind two separate surfaces of two adjacent HA protomers, thereby blocking HA binding to SA (91, 92).

### Conserved protective epitopes on the HA stem domain

In recent decades, tremendous effort has been invested in isolating and structurally characterizing bnAbs that target the HA stem domain. The conserved protective epitopes that these bnAbs recognize are mainly located in two regions: the hydrophobic groove and the fusion peptide. These bnAbs are generally encoded by a relatively restricted set of variable-heavy (VH) gene segments (103). Due to the high conservation of the stem domain, anti-stem bnAbs usually exhibit more widely reactive and neutralizing breadth to influenza A virus strains than anti-head bnAbs. All of the anti-stem bnAbs lack HAI activity. The mechanism of protection involves inhibiting the host cell protease cleavage of HA0 (104–108), or preventing membrane fusion *via* antibody binding to cleaved HA, inhibiting its low pH-induced conformational change (28, 81, 104–114). Anti-stem bnAbs mediating Fc-FcγR responses also play a critical role *in vivo* in the protection (104–106, 110, 115). In addition, antibodies S9-1-10/5-1 and 9H10 inhibit virus particle release from infected cells (114, 116).

#### Epitopes of the hydrophobic groove region

Among the bnAbs that bind to the hydrophobic groove region, some exhibit extremely broad binding properties to all subtype HAs from H1 to H18 (cross-group). Many antibodies exhibit group 1 virus-specific reactivity, whereas only one antibody (SD36) exhibits group 2 virus-specific reactivity (117).

In 2008, antibody CR6261 was the first bnAb reported to exhibit group 1 and group 2 reactivity (31). Since then, a series of this type of bnAb was isolated and identified, such as antibodies FI6(FI6v3) (104), 27F3 (118), 3E1 (119), SD38 (117), 39.29 (120), CT149 (110), 3I14 (105), 31.a.83 (121), 56.a.09 (121), CR9114 (81), MEDI8852 (106), 05-2G02 (122), S9-1-10/5-1 (116), 1.12 (116, 123) and 28-12 (124) (**Table 2**). Most of these antibodies exhibit broad neutralizing activity and protection against influenza A virus *in vivo*.

**Table 2:** Characteristics of broadly neutralizing antibody binding to the HA stem domain.

Location	Antibody name	Binding breadth *in vitro*	Neutralizing breadth *in vitro*	Protection breadth *in vivo*
Hydrophobic Groove Region	FI6(FI6v3)^H^ (104)	H1-H16	H1, H5/H3, H7	H1, H5/H3*
	CR6261^H^ (28, 31, 115)	H1, H2, H5, H6, H8, H9/H7/B	H1, H2, H5, H6, H8, H9	H1, H2, H5
	27F3^H^ (118)	H1, H2, H5, H6, H9, H11, H12, H13, H16/H3, H7, H10/B	H1, H5, H6/H3, H7, H10	–
	3E1^H^ (109, 119)	H1, H5, H9/H3, H7	H1, H5, H9/H3, H7	H1, H5
	SD38^L^ (117)	H1, H2, H5/H3, H7, H10	H1, H2, H5/H3, H7, H10	–
	39.29^H^ (120)	H1, H2, H5/H3, H7	H1, H2/H3	H1, H5/H3*
	CT149^H^ (110)	H1, H5, H9/H3, H7	H1, H5, H9/H3, H7	H1, H5/H3, H7
	3I14^H^ (105, 125)	H1, H2, H5, H6, H8, H9, H11, H12, H16/H3, H4, H7, H10, H14, H15	H1, H5/H3, H7	H5/H3, H7
	31.a.83^H^ (121)	H1, H2, H5, H9/H3, H7	H1, H2, H5, H9/H3, H7	–
	56.a.09^H^ (121)	H1, H5/H3, H7	H1, H5/H3, H7	–
	CR9114^H^ (81, 115)	H1, H2, H5, H6, H8, H9, H12, H13, H16/H3, H4, H7, H10, H15/B	H1, H2, H5, H6, H8, H9, H12/H3, H4, H7, H10	H1, H2/H3/B
	MEDI8852^H^ (106)	H1-H18	H1, H2, H5, H6, H9/H3, H7	H1, H5/H3*
	05-2G02^H^ (122, 126)	H1, H2, H5, H6, H8, H9, H13, H16, H17, H18/H3, H4, H7, H10, H14, H15	H1, H5/H3	H5
	S9-1-10/5-1^H^ (116)	H1-H18	H1, H5/H7	H1, H5/H3, H7
	1.12^H^ (123)	H1, H2, H5, H6, H8, H9, H11, H12, H13, H17, H18/H3, H4, H7, H10, H14, H15	H1-H15	H1/H3
	28-12 ^H^ (124)	H1, H6, H8, H9/H3, H4, H7, H14,	H1/H3, H4, H7	H1/H3
	C179^M^ (111, 127–129)	H1, H2, H5, H6, H9	H1, H2, H5, H6, H9	H1, H5
	F10^H^ (112)	H1, H2, H5, H6, H8, H9, H11, H13, H16	H1, H2, H5, H6, H8, H9, H11	H1, H5
	D8^H^ and A66^H^ (112)	H1, H2, H5, H6, H9, H11, H13, H16	H1, H2, H5, H6, H11	H1, H5
	70-1F02^H^ (126, 130)	H1, H2, H5, H6, H8, H9, H11, H12, H13, H16, H17, H18	H1, H5	H1, H5
	1009-3B05^H^ (126, 130)	H1, H2, H5, H6, H8, H9, H13, H17, H18	H1, H5	H5
	09-3A01^H^ (122, 126)	H1, H2, H5, H6, H8, H9, H13, H16, H17, H18	H1, H5	H5
	Mab3.1^H^ (131)	H1, H2, H5, H6, H18	H1, H2, H5, H6	H1
	A06^H^ (132)	H1, H5	H1, H5	H1
	FE43^H^ (70)	H1, H5, H6, H9	H1, H5, H6, H9	H1, H5, H6
	4C2^M^ (133)	H1, H2, H5, H9	H1, H2, H5, H9	H1
	1H11^H^ and 5G2^H^ (113)	H1, H5, H9	H1, H5, H9	–
	2H5^H^ (113)	H1, H5, H9	H1, H5	–
	SD36^L^ (117)	H3, H4, H7, H10	H3, H4, H7, H10	–
	SD83^L^ (117)	B	B	–
Fusion Peptide Region	CR8020^H^ (107)	H3, H4, H7, H10, H14, H15	H3, H7, H10	H3, H7
	CR8043^H^ (108)	H3, H4, H7, H10, H14, H15	H3, H10	H3, H7
	9H10^M^ (114)	H3, H10	H3, H10	H3

^H^ human antibody; ^M^ murine antibody; ^L^ llama antibody; B, influenza B viruses; -, no information.

Binding breadth in vitro, cross-react with expressed different HA proteins or viruses *in vitro*.

Neutralizing breadth in vitro, effectively neutralize and cross-neutralize different influenza viruses in cells.

Protection breadth in vivo, effectively preventing and/or therapeutic efficacy against influenza virus infection in mouse animal models, with an exception “*” mouse and ferret were used as animal models.

**Figure 3:**
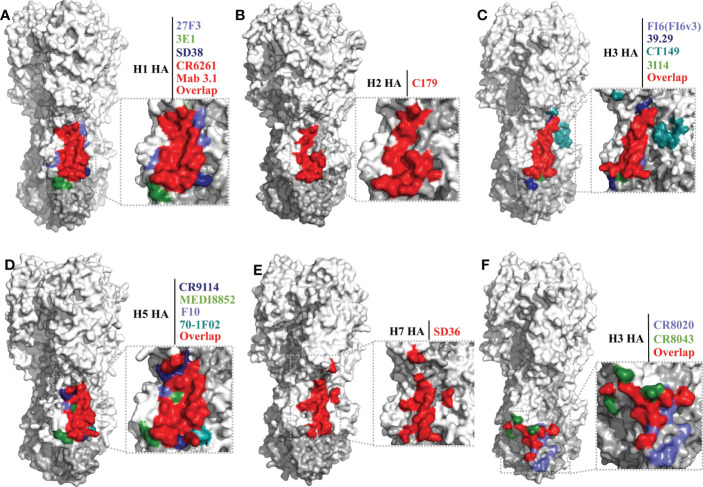
Conserved epitopes of the HA stem domain. HA1 is shown in white, HA2 in gray. **(A–E)** are epitope footprints of bnAbs binding the hydrophobic groove region. **(A)** H1 HA (PDB ID: 4M4Y) as a model, the epitopes of CR6261 (PDB ID:3GBN) and Mab 3.1 (PDB ID: 4PY8) overlap in red. The non-overlapping residues are shown in light purple (27F3, PDB ID: 5WKO), green (3E1 PDB ID: 5GJT) and dark blue (SD38, PDB ID: 6FYT), respectively. **(B)** H2 HA (PDB ID: 4HLZ) as a model, the epitope of C179 (PDB ID: 4HLZ). **(C)** H3 HA (PDB ID: 4FNK) as a model, the non-overlapping residues are shown in light purple (FI6 (FI6v3), PDB ID: 3ZTJ), dark blue (39.29, PDB ID: 4KVN), blue-green (CT149, PDB ID:4UBD), and green (3I14, PDB ID: 6WF0), respectively. **(D)** H5 HA (PDB ID: 4MHH) as a model, the non-overlapping residues are shown in dark blue (CR9114, PDB ID: 4FQI), green (MEDI8852, PDB ID: 5JW4), light purple (F10, PDB ID: 3FKU), and blue-green (70-1F02, PDB ID: 6B3M), respectively. **(E)** H7 HA (A/tree sparrow/Shanghai/01/2013) as a model, the epitope of SD36 (PDB ID: 6FYU). **(F)** Epitope footprint of bnAbs binding to the fusion peptide region. H3 HA (PDB ID: 4FNK) as a model, the non-overlapping residues are shown in light purple (CR8020, PDB ID: 3SDY) and green (CR8043, PDB ID: 4NM8).

The epitope footprints of antibodies CR6261, 27F3, 3E1, SD38, and Mab 3.1 are shown in the H1 HA model of **Figure 3A**. All of these antibodies bind to the hydrophobic groove region with CDRs of heavy and light chains. Antibody 3E1 also bind to the fusion peptide region (109, 119). CR6261 neutralizes viruses by blocking conformational rearrangements of HA associated with membrane fusion (28, 31, 115). The epitope footprints of antibodies FI6(FI6v3), 39.29, CT149, and 3I14 are shown in the H3 HA model of **Figure 3C**. FI6(FI6v3) inhibits the conformational changes of HA, prevents membrane fusion, and inhibits HA0 processing (104). CT149 binds residues of two adjacent protomers of HA, and CT149 and 3I14 neutralize viruses by inhibiting low pH-induced membrane fusion (105, 110). The epitope footprints of antibodies CR9114 and MEDI8852 are shown in the H5 HA model of **Figure 3D**. CR9114 exhibits extremely broad reactivity, including against 14 influenza A subtypes and both influenza B virus lineages, and it can neutralize 11 of these subtypes of virus. CR9114 also protected mice from lethal challenge with H1N1, H2N2, H2N3, H3N2, and influenza B viruses in prophylaxis studies (81, 115). MEDI8852 binds to the hydrophobic groove and a large portion of the fusion peptide through a coordinated movement of CDRs (106).

Antibody C179 was the first reported bnAb to neutralize influenza A viruses, and was isolated from a mouse in 1993 (111). In the last decade, many bnAbs with group 1 virus-specific reactivity targeting the hydrophobic groove region have been isolated, including antibodies F10 (112), D8 (112), A66 (112), 70-1F02 (130), 1009-3B05 (130), 09-3A01 (122), Mab3.1 (131), A06 (132), FE43 (70), 4C2 (133), 1H11 (113), 5G2 (113), and 2H5 (113) (**Table 2**). C179 cross-neutralizes H1, H2, and H5 subtype viruses, and protects mice from lethal challenge with H5N1 and 2009 H1N1 pandemic viruses (111, 127, 128). The epitope of C179 bound to the 1957 H2N2 HA is similar to that of bnAbs CR6261, F10, CR9114, and FI6(FI6v3) (**Figures 3A–D**) (127). F10 bound to H5 HA with heavy-chain CDRs inserts into the hydrophobic groove pocket and locks the fusion peptide (112) (**Figure 3D**).

As mentioned previously, of the bnAbs that bind to the hydrophobic groove region, only one exhibits group 2 HA reactivity, the llama single-domain antibody (sdAb) SD36 (**Table 2**). SD36 recognizes conserved epitopes that partially overlap with those of bnAbs CR9114, CR6261, and FI6(FI6v3) (117) (**Figures 3A, C–E**). Why are bnAbs with group 2 virus-specific reactivity that target the hydrophobic groove region so rare? It may be that a conserved Asn38 glycan in the HA1 of group 2 viruses may interfere with the accessibility of the conserved antigenic site on helix A (28, 112, 134). However, some antibodies, such as 27F3, can navigate around this N38 glycan to achieve cross-group neutralization (118, 135).

Llama sdAb SD83 can neutralize both influenza B virus lineages (**Table 2**), and its epitope is also in the hydrophobic groove region. This epitope is highly conserved, with the residues being >99% identical in influenza B viruses (117).

Till now, a series of small protein or peptide viral fusion inhibitors targeting conserved epitopes of HA stem have been developed, such as HB36 and HB80 (19), HB36.3 and HB80.4 (23), HB36.6 (21), JNJ4796 (22), P7 (20). Some of these inhibitors have been shown to protect mice from influenza virus infection, such as HB36.6 and JNJ4796 (21, 22), and four antibody drugs (CR6261, 39.29, CR8020 and MEDI8852) have entered phase II clinical trial (136–138). In addition, several universal vaccines based on the HA stem, such as headless HA and HA mini-stems, have also shown promising prospects, and chimeric HA has entered phase I clinical trial (136, 137).

#### Epitopes of the fusion peptide region

The bnAbs that bind to the fusion peptide region of the HA stem are only reactive with group 2 HA; these bnAbs include CR8020 (107), CR8043 (108), and 9H10 (114) (**Table 2**). The epitopes recognized by these antibodies are located lower down on the stem domain, close to the virus membrane, but accessible on virions (**Figure 3F**). Electron microscopy reconstructions shows that the three antibodies bind to a similar epitope footprint, but their sensitivities to mutations are distinct, due to their slightly different approach angles to the HA (114). These antibodies inhibit viral replication by blocking membrane fusion and mediating Fc-FcγR responses. In addition, CR8020 inhibits the host cell protease cleavage of HA0 (107) and 9H10 disrupts viral particle egress in the late stage of infection (114).

**Figure 4:**
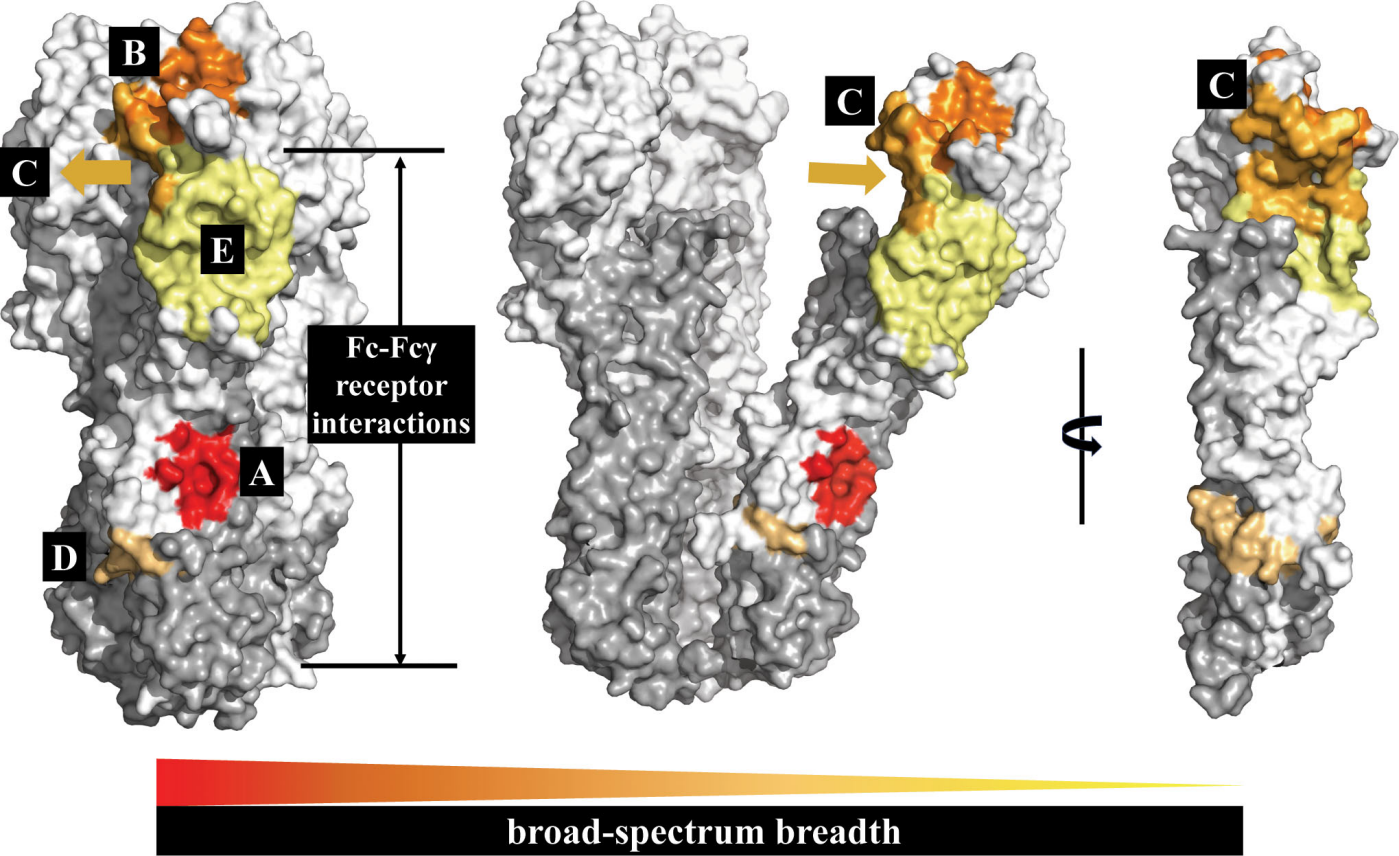
The HA conserved epitope regions of influenza A virus. HA1 is shown in white, HA2 in gray. The five conserved protective epitope regions summarized in this review: **(A)** The hydrophobic groove region is shown in red; **(B)** the RBS in orange; **(C)** the occluded epitope region of the HA monomers interface in light orange; **(D)** the fusion peptide region in orange-yellow; and **(E)** the VE subdomain in yellow. The broad-spectrum breadth of the five conserved protective epitope regions are ranked A>B>C>D>E.

In summary, the highly conserved protective epitopes of HA in influenza A viruses are concentrated in five regions: the RBS pocket, the VE subdomain, the occluded epitope region between the HA heads, the hydrophobic groove region, and the fusion peptide region. The breadth of bnAbs targeting these five conserved protective epitope regions is summarized in **Figure 4**. The hydrophobic groove region is the most conserved protective epitope region of HA. Most bnAbs targeting these regions exhibit extremely broad reactivity to both group 1 and/or group 2 HAs of influenza A virus, and even to influenza B virus. The epitopes of the RBS pocket and the occluded epitope region are also conserved; some bnAbs targeting these two regions are broadly reactive, but bnAbs targeting the RBS pocket having higher potency than bnAbs targeting the occluded epitope region. To date, bnAbs targeting the fusion peptide region only neutralize group 2 HAs, and bnAbs targeting the VE subdomain are subtype-specific.

## Conclusions

The constant antigenic drift and antigenic shift of influenza viruses and the outbreak of the SARS-CoV-2 pandemic since 2020 have further emphasized the urgent need for a universal influenza vaccine and therapeutic agents. The isolation of new bnAbs and identification of highly conserved protective epitopes of HA have identified more distinct targets for the development of novel vaccines and therapeutic based on HA. Because the epitopes of the HA head are more accessible, humoral responses to the head region of HA are more robust than those to the HA stem. In one study, about 14% of HA-specific memory B cells from healthy human donors, 76% B cell receptor were specific for epitopes present on the HA head (69). Generally, antibodies targeting the head of HA shown more potency by directly or indirectly blocking viral adsorption (61, 74). Such antibodies can neutralize infectious viruses at a low concentration, in contrast with antibodies to the stem (70, 112). So, the characteristics of the conserved protective epitopes on the HA head are more suited to the requirements of targets of novel vaccine development than those on the stem. The antibodies that target the stem of HA offer broader but less potent reactivity. The epitopes of HA stem are intrinsically less permissive for mutations, due to the need to maintain interchain packing and to undergo conformational changes during the fusion process (139). So, the characteristics of the epitopes on the HA stem are still compatible with therapeutic development.

In recent years, significant advances have been made in universal influenza vaccine research, and multiple strategies are currently being explored based on HA, including chimeric HA, mosaic HA, computationally optimized broadly reactive antigens (COBARs), Mini-HA, and mosaic nanoparticle vaccination approaches (140, 141). To date, series of universal influenza vaccine (136, 142, 143) and therapeutic agents (137, 144) that targeting HA have been tested in clinical trials. We are confident that as the highly conserved protective epitopes of HA are more clearly elucidated and the antiviral mechanism of bnAbs becomes clearer, universal influenza virus vaccines that provide higher vaccine efficacy and more novel therapeutic candidates that mimic the function of bnAbs will be developed.

## Author contributions

All authors listed have made a substantial, direct, and intellectual contribution to the work, and approved it for publication.
